# Use of tumor treating fields for a malignant brain tumor in a pregnant woman: Case report

**DOI:** 10.1016/j.bas.2026.105973

**Published:** 2026-02-08

**Authors:** Lucas Rubisoier, Leonardo Lustgarten, Claudius Thomé, Christian F. Freyschlag, Johannes Kerschbaumer

**Affiliations:** a Department of Neurosurgery, Medical University of Innsbruck, Innsbruck, Austria; b Novocure Inc, New York, NY, USA

**Keywords:** TTF therapy, Optune-Gio, Pregnancy, Anaplastic ganglioglioma, Safety

## Abstract

**Introduction:**

Brain tumors diagnosed during pregnancy are exceptionally rare, and their clinical progression is not yet fully understood. Managing intracranial tumors during pregnancy necessitates a specialized approach, balancing neuro-oncological considerations with obstetric concerns to evaluate therapeutic options effectively.

Tumor Treating Fields (TTF) therapy is a non-invasive, regionally-applied, anti-cancer treatment modality approved for adults with newly diagnosed and recurrent glioblastoma. To date, there is no data on the safety and efficacy of TTF therapy in pregnant patients harbouring malignant brain tumors.

**Research question:**

To describe a case of TTF-use during pregnancy and provide a first insight into its feasibility.

**Materials and methods:**

We describe the first reported case of a pregnant women treated with TTF. Medical records and imaging data were analysed. Relevant literature concerning the management of malignant brain tumors in pregnancy was reviewed.

**Results:**

The patient diagnosed with a posterior fossa high-grade anaplastic ganglioglioma (CNS WHO grade 3-4) received surgical resection followed by radiotherapy with fetal shielding combined with TTF therapy during pregnancy.

**Discussion and conclusion:**

Since TTF therapy represents a topical treatment without systemic application, we suggest it as a feasible option for pregnant patients diagnosed with malignant brain tumors.

## Highlights

• Malignant brain tumors diagnosed during pregnancy pose unique challenges.
• Tumor-treating fields (TTF) offer a possible treatment option among limited alternatives.
• We present the first reported use of TTF in a pregnant patient.

## 1 Introduction

Brain tumors diagnosed during pregnancy are fortunately very rare. They represent a life-threatening disease (Buckner et al., 2007), and the condition of both the mother and fetus must be considered during pregnancy. Because of the low prevalence, there are no clear guidelines on treatment regimens in these situations. The decision on whether brain tumors should be treated during pregnancy or after termination of pregnancy is debatable, depending on suspected brain tumor diagnosis, maternal wishes plus condition and gestational age of the fetus.

The incidence of primary malignant brain tumors in pregnant women is roughly the same as in non-pregnant women in the same age group (Somma et al., 2023). Intracranial tumors occur in 2.6 per 100,000 pregnant women in the United States, with glioma being the most prevalent histological type (Yust-Katz et al., 2014). Due to this low incidence, evidence on clinical outcome and management of pregnant glioma patients is based on small case series and expert opinions. Previous studies have provided potential management algorithms for gliomas in pregnancy, but the evidence is sparse, and many questions remain unanswered (Peeters et al., 2018; Tewari et al., 2000).

Being faced with the diagnosis of a brain tumor during pregnancy surely poses an enormous challenge for the expectant mother. Additionally, the situation can also be demanding for the treating team of healthcare providers due to limited treatment options. For high-grade gliomas (CNS WHO grade 4), chemoradiation followed by chemotherapy is the standard of care (Fisher and Adamson, 2021). In selected patients, surgery can be safely performed at a later stage of pregnancy and followed by focal cranial irradiation with the use of a shielding device to minimize radiation exposure to the fetus (Yust-Katz et al., 2014; Horowitz et al., 2014). During pregnancy, chemotherapy is contraindicated due to its teratogenic effects (Van Westrhenen et al., 2018). While there are concerns on the use of magnetic resonance imaging (MRI) during the first trimester, MRI is considered a safe imaging modality in the second and third trimester (Lum and Tsiouris, 2020). However, the use of gadolinium-based contrast is often determined on a case-by-case basis after completion of the first trimester due to unknown risks to the fetus (Fraum et al., 2017).

Given the lack of definitive data to formulate standard treatment guidelines, developing individualized treatment plans is necessary for glioma patients who are pregnant. Clinical factors to consider when developing such treatment plans include tumor grade, extent of resection, and gestational status. Another essential factor to consider is the wishes of the pregnant patient with regards to her baby.

Tumor Treating Fields (TTF) therapy is a non-invasive, loco-regionally applied, anti-cancer treatment modality approved for adults with newly diagnosed and recurrent glioblastoma (GBM) (Stupp et al., 2012, 2017). TTF are low intensity intermediate frequency, alternating electric fields generated by a portable medical device and are delivered noninvasively to the tumor site via arrays placed on the skin. The electric fields act selectively on cancer cells due to their unique properties and exert physical forces to disrupt cellular processes crucial for cell viability and progression (Kirson et al., 2004; Voloshin et al., 2020; Karanam and Story, 2021).

The first approval for TTF therapy was granted by the FDA (Food and Drug Administration) in 2011 for the treatment of recurrent GBM, following the results of the randomized, pivotal (phase 3) EF-11 clinical study (Stupp et al., 2012). In 2015, additional approval was granted based on results of the randomized, pivotal (phase 3) EF-14 clinical study for TTF therapy in newly diagnosed GBM with maintenance temozolomide after standard chemoradiation. EF-14 demonstrated statistically significant improvement in overall survival and long-term (5-year) survival vs temozolomide alone (Stupp et al., 2015, 2017). TTF therapy usage has been shown to positively correlate with increased survival outcomes (Toms et al., 2019; Ballo et al., 2023).

Currently, regulatory labelling states that TTF therapy use should be avoided in patients who are pregnant or trying to get pregnant due to insufficient data as TTF treatment has not been tested in pregnant women and further research would be necessary to assess safety and tolerability in this particular patient population.

Here, we present the case of a pregnant woman with a large posterior fossa anaplastic ganglioglioma who underwent surgery, radiotherapy and TTF. This case provides unique insights on the course of a pregnant patient including the feasibility of the clinical application of TTF therapy during pregnancy.

## 2 Case report

We present the case of a 35-year-old patient, who arrived at the emergency department with a referral from her obstetrician. She had an intact pregnancy at 12 weeks and 5 days of gestation and symptoms including headaches, dizziness, blurred vision and unsteady gait for the past 4 weeks. Additionally, she complained of right-sided hearing loss in the days prior to admission. The patient was Gravida 1, Para 0 and previously well and without any risk factors or family history of CNS cancers.

Upon examination at presentation, she showed a spontaneous linear rotating nystagmus to the right side which increased upon right-sided gaze and use of Frenzel goggles and subsided upon left-sided gaze. A positive head impulse test on both sides as well as right-sided hearing loss and gait imbalance with lateropulsion to the left were also noticed. Unterberger stepping test caused rotation to the left. Otherwise, the neurological examination revealed no pathological findings, especially no cranial nerve palsy.

**Fig. 1:**
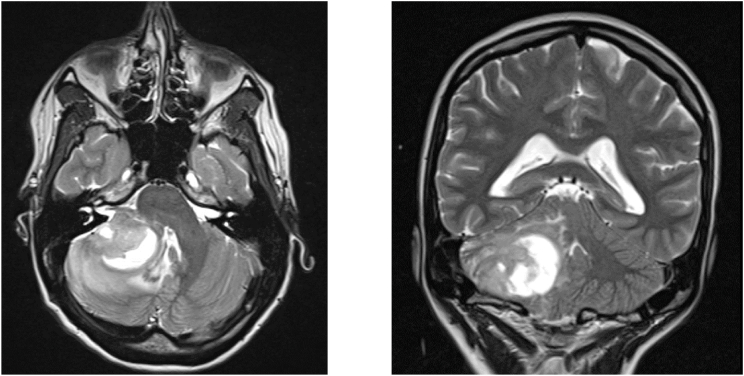
Brain MRI without contrast at admission, axial (left) and coronal (right) T2 sequence.

Given her clinical presentation, a brain MRI was performed without administration of contrast-agent (due to pregnancy), revealing a significant posterior fossa mass in the right cerebellopontine angle which seemed to infiltrate the right cerebellum (see Fig. 1). The initial radiological differential diagnosis included an intra-axial tumor or a cystic vestibular schwannoma. The lesion caused a compression of the fourth ventricle without hydrocephalus. The patient was admitted to the neurological intensive care unit for further diagnostic workup and surgical planning.

She received 8mg of i.v. dexamethasone daily which improved her symptoms and she could be transferred to the general neurological ward the next day. Gynecology was consulted before surgery and ultrasound showed normal development of the fetus without pathological findings.

She underwent surgery a week after admission, via a suboccipital approach using intraoperative neuromonitoring. The surgical procedure went uneventful. Intraoperatively, the tumor was found to be infiltrating the right cerebellar hemisphere and tonsil and the frozen section analysis revealed a malignant primary brain tumor. Because the caudal cranial nerves were surrounded by the tumor mass, a subtotal resection was achieved -sparing the cranial nerves to reduce the risks of dysphagia and dyspnea. The intraoperative neuromonitoring detected no abnormalities.

Postoperatively the nystagmus remained, and she developed a mild bilateral palsy of the VI. cranial nerve, which improved quickly. A phoniatric consultation also diagnosed mild dysphagia which was treated by speech-language therapists.

**Fig. 2:**
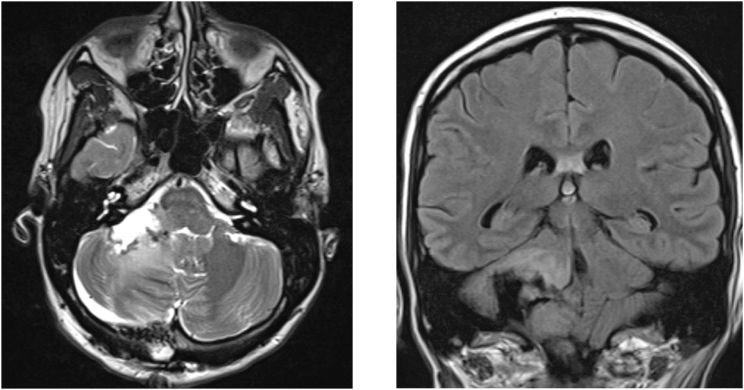
Postoperative MRI without contrast, T2 axial (left) and T2 TIRM coronal (right).

Her postoperative brain MRI confirmed the subtotal resection (see Fig. 2), and the postoperative course was otherwise uneventful. She was discharged home on the 11th postoperative day (15 weeks of gestation) with mild cerebellar symptoms (unsteady gait, dizziness). An ultrasound at the time of discharge showed a normal pregnancy with fetal movement and heart activity detected.

**Fig. 3:**
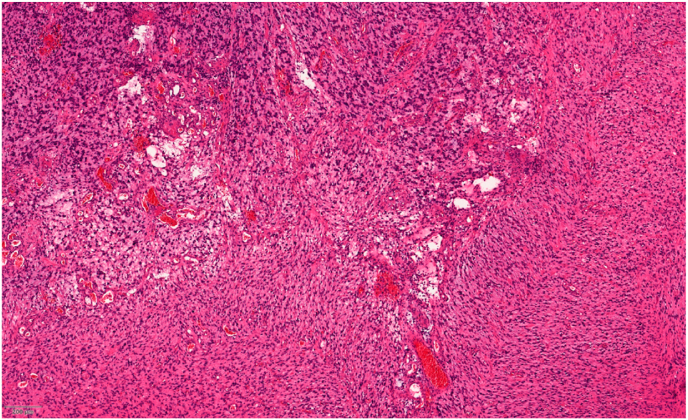
H&E-stained histology slide of the patient's tumor (100x).

The integrated histologic-molecular diagnosis of the pathology report described an anaplastic ganglioglioma CNS WHO grade 3-4, IDH1/2 wild type, with loss of ATRX-protein expression, BRAF negative, H3F3A negative, HIST1H3B negative, and MGMT moderately methylated (21%). MIB1 was positive in 20% (see Fig. 3). Furthermore, a methylation analysis was added, which classified the tumour as an anaplastic pilocytic astrocytoma (Heidelberg classifier calibrated score 0.992) with homocygotic loss of CDKN2A.

Treatment options were discussed with the patient. Given the IDH wildtype tumor with high proliferation rate, a treatment regime similar to that used for GBM was suggested. Because adjuvant chemotherapy with temozolomide would have required the pregnancy to be terminated, the patient decided against administration until birth of the baby. Instead, radiotherapy was initiated with abdominal shielding of the fetus. She received a total radiation dose of 54.0 Gy to the resection cavity, with 1.8 Gy daily fractions for 6 weeks.

TTF were suggested to the patient as an off-label treatment that could be used in her case. This was proposed due to the known survival benefit of TTF in GBM as well as the similar biological characteristics of the present tumor compared to GBM. Due to the effect of TTF confined to the brain, adverse effects to the fetal development were considered unlikely and therapy was started.

The fetus was delivered by primary cesarean section at 36 weeks of gestational age, 5 months after initial diagnosis. The newborn girl weighed 2495g at birth, had a length of 46cm and an Apgar-Score of 6/9/10. As the newborn and mother were well, both were discharged early. TTF were interrupted for 3 days during the immediate postpartum period.

**Fig. 4:**
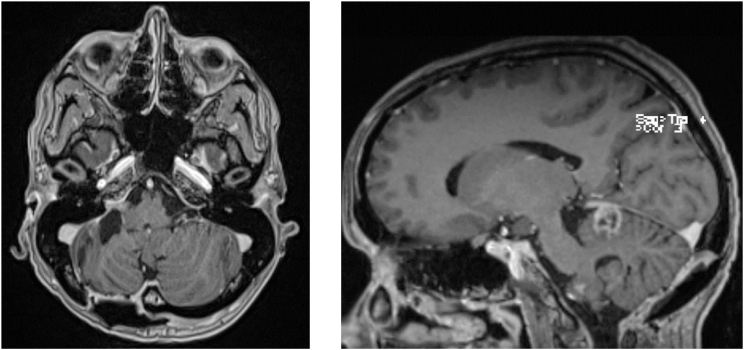
Postpartal MRI T1 with contrast depicting stable tumor at the resection cavity (left) and a second infratentorial lesion (right).

Upon discharge, an MRI with contrast enhancement was performed (see Fig. 4) to plan future oncological treatment. Upon clinical evaluation, she had mild dizziness that did not impair her mobility. The MRI showed both the known residual tumor and a second focus close to the tentorium with marginal contrast-enhancement. Both tumor components were stable when compared to the immediate postoperative scans, which were deemed as satisfactory when considering the limited oncologic therapy options. Because of the tumor's positive MGMT-methylation status, temozolomide therapy was started 5 months after diagnosis with cessation of breastfeeding.

She went on to receive a total of 6 adjuvant cycles of temozolomide. The 2nd and 3rd cycle of temozolomide were prematurely cancelled due to vasovagal syncopes and grand mal seizure.

Due to newly developed seizures and episodic confusion, cerebrospinal fluid (CSF) samples and another MRI were obtained 7 months after surgery. An increasing enhancement of the pituitary stalk on the MRI was suggestive of leptomeningeal disease. While CSF microscopy showed no malignant cells, protein levels and albumin CSF/serum ratio were also increased. Therefore, bevacizumab was administered in combination with temozolomide. During this time period, TTF were paused by the patient for 2 months.

**Fig. 5:**
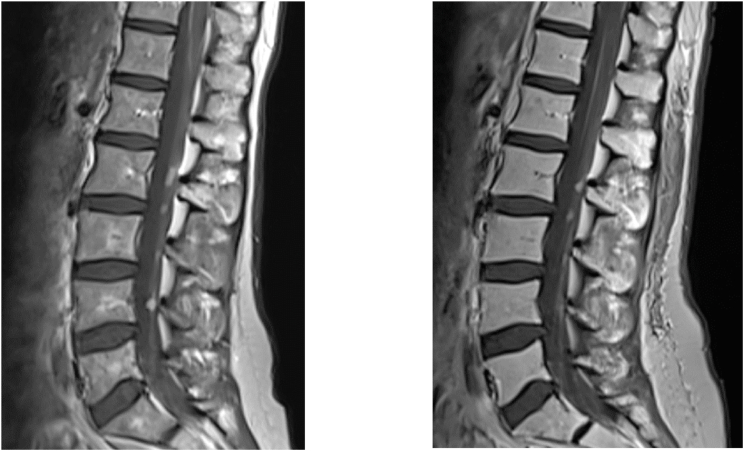
Extramedullary spinal metastases on sagittal T1 MRI with contrast. Before (left) and 6 months after radiotherapy (right).

Additionally, MRI images of the spine were performed (see Fig. 5) which identified multiple extramedullary metastases in the lumbar, thoracic and cervical spine as a spinal extension of the leptomeningeal disease. However, at this point in time, the patient was completely asymptomatic for the metastases. At initial presentation, no spinal imaging was performed due to a lack of symptoms suggesting a spinal pathology. Following this diagnosis, whole spinal axis radiation therapy was initiated (39.6 Gy in fractions of 1.8 Gy over a course of 4 weeks) and TTF were continued. The radiotherapy was tolerated well and shrinkage of the spinal metastases was noted on subsequent MRIs.

**Fig. 6:**
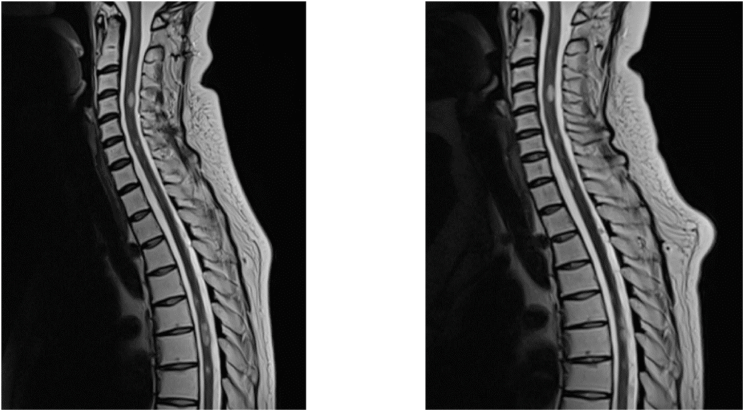
Intramedullary metastases leading to paraparesis in the patient. Sagittal T2 MRI of the cervical and upper thoracic spine before (left) and after radiotherapy (right). A slight reduction in metastasis size was achieved at the Th6 level.

Later, 20 months after diagnosis, the patient developed progressive paraparesis due to intraspinal metastases. Regardless of immediate initiation of palliative radiotherapy (25 Gy in fractions of 2.5 Gy) the patient remained immobile from this point forward. The site of the primary tumor remained controlled, but the patient succumbed to the disseminated disease two years and 9 days after initial diagnosis (see Fig. 6).

**Fig. 7:**
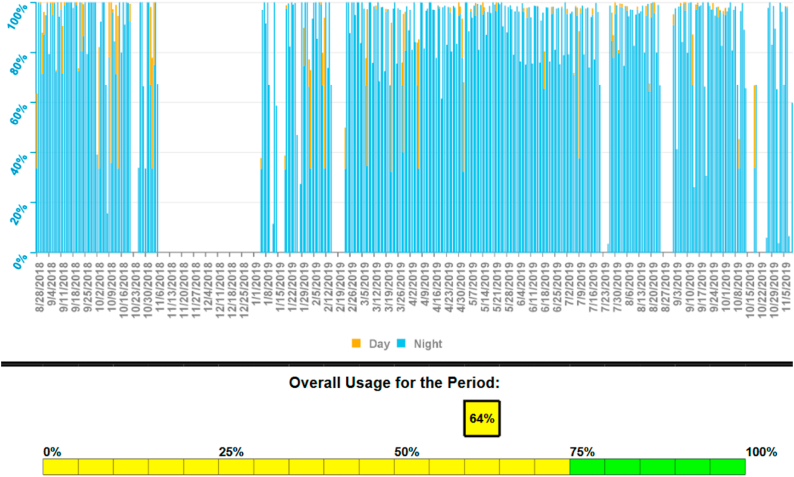
Overall usage of TTF in the described patient. The recommended overall usage of 75% was not reached due to a 2-month period during which the patient did not use TTF at all.

TTF were administrated over a period of 16 months and were interrupted for 3 days postnatal and for a 2-month period during which the patient was unwell due to seizures and syncopes The patient did not report any side effects associated with the therapy such as skin irritation, redness or headaches. The overall usage data is represented in Fig. 7.

## 3 Discussion

To the best of our knowledge, this represents the first ever reported case on the use of TTF in a pregnant patient.

Management of intracranial tumors presenting during pregnancy requires a complex decision-making process using a multidisciplinary team determining whether to operate as soon as the tumor is discovered or to schedule fetal delivery with delayed surgical management. From a neurosurgical perspective, the decision is influenced by the tumor's suspected biology, location, growth rate, and size. Malignant and rapidly growing tumors in eloquent areas require aggressive treatment and timely surgical intervention. Another important factor is the patient's clinical and neurological status.

Suspected high-grade gliomas diagnosed during pregnancy may be surgically removed at presentation, but attempts should be made to delay surgery and delivery until fetal maturity in the early third trimester. In these cases, delayed surgical management can be considered with continued close obstetrical monitoring (Van Westrhenen et al., 2018), however, there is a risk for maternal and fetal life due to raised intracranial pressure (ICP). Induction of labor may be considered as early as 34 weeks in asymptomatic patients followed by surgical resection of the tumor (Johnson et al., 2009).

Should the maternal neurologic condition be unstable or if the tumor is diagnosed early in pregnancy (and thus delaying surgery until fetal maturity seems improbable) it may be necessary to carry out the resection during pregnancy. In these cases, the second trimester seems to be the most favorable for surgery (Van Westrhenen et al., 2018). During the first trimester the fetus is more vulnerable and in the third trimester the risk of intraoperative hemorrhage is raised due to an increase in maternal intravascular volume. When the fetus is viable and maternal deterioration due to raised ICP occurs, an emergency cesarean section should be performed (Tewari et al., 2000; Van Westrhenen et al., 2018).

Based on currently available data, there remains little guidance on the management of intracranial tumors during pregnancy. The best recommendation is to discuss each case individually with its own assessment of the risks and benefits of early versus late surgical intervention.

In general, the treatment options for malignant brain tumors during pregnancy are the same as the standard available choices. However, due to the associated toxicities and potential teratogenic effects, chemotherapy and radiotherapy need to be considered on a case-by-case basis (depending on the drug and trimester).

The European Society of Medical Oncology clinical practice guidelines on systemic therapy in pregnancy recommends avoiding chemotherapy during the first trimester due to the high risk of congenital malformations in nearly 20% of patients (Peccatori et al., 2013). Those with aggressive malignancies needing chemotherapy in the first trimester should consider termination of pregnancy. Even in later trimesters, chemotherapy should be used very restrictively and must be assessed based on the individual agent and situation (Peccatori et al., 2013). Temozolomide, an alkylating agent used as standard of care for the first line treatment of malignant gliomas, is not advised for use in pregnant women. Although there have been case reports of pregnant women receiving temozolomide during the first weeks of pregnancy and delivering healthy newborns (Blumenthal et al., 2008).

Radiotherapy delivery during pregnancy requires meticulous considerations of all alternative options. For high-grade gliomas, adjuvant radiotherapy improves survival and probably should not be delayed. If malignancy is diagnosed in the early first trimester, a therapeutic abortion should be considered to enable the entire treatment course of chemoradiation. Adjuvant radiotherapy (starting within 4 weeks of surgery) can be offered in any trimester with all efforts to minimize fetal dose. Especially during the first trimester, therapeutic cerebral irradiation can cause serious harm to the fetus and should, therefore, be avoided (Horowitz et al., 2014; Johnson et al., 2009; Haba et al., 2004; Lew et al., 2010). Radiation doses >50 Gy pose a risk to the fetus during all trimesters (Blumenthal et al., 2008). It has been advised to calculate and minimize the scattered dose and additional shielding of the pelvis (Tewari et al., 2000; Blumenthal et al., 2008; Haba et al., 2004).

With the limited treatment options available for these patients and the potential teratogenic effects of chemotherapy on the fetus, alternative therapeutic approaches are essential. The use of TTF presents a promising option due to its loco-regional delivery and the absence of systemic toxicity. This case report aims to provide insight on the feasibility of using an already approved treatment for malignant tumors during pregnancy.

While there is no data on the use of TTF in pregnant patients, data on the pediatric population is also limited. Preclinical studies have showed an effect of TTF on cell lines of pediatric brain tumors (Nitta et al., 2022; Branter et al., 2022). Furthermore, although off-label, TTF were used in pediatric patients and are considered a generally safe and feasible treatment option in underage patients (Rousseau et al., 2025; Goldman et al., 2022).

While higher usage time of TTF is associated with longer progression-free survival (Deguchi et al., 2025), experience outside of clinical trials indicates that the average usage time is around 65% (Jelgersma et al., 2024). This shows that in a majority of patients, the goal of 75% is not met. Factors leading to poor therapy adherence, such as skin irritation or skepsis regarding its effectiveness, should therefore be identified and addressed. In the case presented here, the overall usage was 64%, also falling short of the intended usage time. This was mainly caused by a 2-month pause due to a phase of poor general condition of the patient (most likely because of leptomeningeal spread which was diagnosed shortly afterwards).

The interpretation of case studies is limited as they represent an individual patient's experience only, and the findings may not be generalizable to broader patient populations. In our presented case, the site of surgical resection remained controlled during treatment with TTF in combination with radiotherapy and subsequent chemotherapy. Still, the patient passed away due to the metastatic spread of the disease. Ultimately, the course without the administration of TTF remains unknown. This case report represents the difficult challenges of treating a malignant brain tumor during pregnancy with extremely limited treatment options. To improve management of this important patient population, prospective data would be beneficial in the future.

## Conflict of interest

The authors declare the following financial interests/personal relationships which may be considered as potential competing interests:Leonardo Lustgarten reports a relationship with Novocure Inc that includes: employment and equity or stocks. Christian Freyschlag reports a relationship with Novocure Inc that includes: consulting or advisory and speaking and lecture fees. Johannes Kerschbaumer is a reviewer for Brain&Spine. Given his role as reviewer, he had no involvement in the peer review of this article and had no access to information regarding its peer review. Full responsibility for the editorial process for this article was delegated to another journal editor."

Christian Freyschlag is a reviewer for Brain&Spine. Given his role as reviewer, he had no involvement in the peer review of this article and had no access to information regarding its peer review. Full responsibility for the editorial process for this article was delegated to another journal editor."

If there are other authors, they declare that they have no known competing financial interests or personal relationships that could have appeared to influence the work reported in this paper.
